# Association between physical activity and sleep-disordered breathing in male Japanese workers: a cross-sectional study

**DOI:** 10.1186/s13104-016-2362-2

**Published:** 2017-01-09

**Authors:** Hiroaki Itoh, Kazuhito Yokoyama, Takehisa Matsukawa, Fumihiko Kitamura

**Affiliations:** Department of Epidemiology and Environmental Health, Juntendo University Faculty of Medicine, 2-1-1 Hongo, Bunkyo-ku, Tokyo, 113-8421 Japan

**Keywords:** Physical activity, Physical exercise, Sleep apnea, Epidemiology

## Abstract

**Background:**

Whether physical activity reduces the risk of sleep-disordered breathing (SDB) for non-obese people remains unclear. The present cross-sectional study examined the association between physical activity and SDB among non-obese male Japanese workers.

**Methods:**

All 200 workers in a company in Tokyo, Japan, who drove a motor vehicle as part of their job, were invited to be screened for SDB to prevent traffic accidents. Of these, 195 agreed to participate in this study. The number of apnea and hypopnea episodes occurring during one night was measured using a single-channel airflow monitor to obtain an individual respiratory disturbance index (RDI). SDB was defined as RDI ≥15 apneas/hypopneas/h. Non-obese males (body mass index <30 kg/m^2^) were included in the analysis. Unconditional logistic regression analysis was used to calculate crude and adjusted odds ratios (ORs) and 95% confidence intervals (CIs) for SDB by physical activity level tertile, as measured by the International Physical Activity Questionnaire.

**Results:**

The prevalence of SDB was 26.9%. The unadjusted analysis showed a significant inverse association between physical activity and SDB: crude ORs for the tertiles of physical activity were 1.00 (low), 1.58 (middle), and 0.27 (high) (95% CI 0.08–0.88; *P* for trend = 0.007). However, this association was attenuated after adjusting for covariates: Adjusted ORs were 1.00 (low), 1.65 (middle), and 0.41 (high) (95% CI 0.10–1.61; *P* for trend = 0.11).

**Conclusions:**

In a cross-sectional study among non-obese male workers in Japan, we found no significant association between physical activity and SDB.

## Background

Physical activity has been hypothesized to have a protective effect against the development of sleep-disordered breathing (SDB). There are several possible mechanisms by which physical activity is thought to mitigate SDB, such as through the maintenance of body weight [1], possible influence of muscle tone in the neck [2] or the influence on visceral body fat [3].

A few previous epidemiologic studies in the United States have suggested a reduced risk of SDB in association with physical activity [1, 4–6]. A longitudinal study showed that additional adjustment for body mass index (BMI) attenuated the association [1]. This suggests that BMI may be a mediator of this effect. A cross-sectional study also showed that the protective association between physical activity and SDB was seen mainly in males [5]. However, these studies investigated the association of physical activity with SDB among predominantly obese or overweight populations (mean BMI 27–32.5 kg/m^2^).

Little evidence is available from epidemiologic studies regarding the association between physical activity and SDB among less obese people. Additionally, to our knowledge, this association has not yet been studied outside the United States. It has been reported that Far East Asian male patients with obstructive sleep apnea, which accounts for the majority of SDB, were less obese but had greater severity of obstructive sleep apnea when compared with white male patients with obstructive sleep apnea [7]. However, Asian facial/mandibular shape may have contributed to these results [8]. Therefore, it remains to be elucidated whether physical activity reduces the risk of SDB in non-obese people, particularly in Asian countries. Weight change, and the general strengthening and fatigue resistance of the ventilatory and upper airway dilator muscles caused by physical activity may improve SDB [3] even for non-obese people.

The present study used a cross-sectional design to examine the association between physical activity and SDB among non-obese male workers in Japan. Our investigation among this group will be very significant for Japanese society in terms of driving and road safety.

## Methods

### Participants

A cross-sectional sample of subjects participated in this study. In October and November 2013, all 200 workers from a nationwide petroleum-related company in Tokyo, Japan, who drove a motor vehicle as part of their job were invited to participate in SDB screening to prevent possible traffic accidents. Of these, 195 agreed to participate in the present study (participation rate: 97.5%). Written informed consent was obtained from all participants. To avoid confounding by sex, female participants (n = 2) were excluded from the analysis. Accordingly, all further analyses were restricted to male participants. The study protocol was approved in advance by the Institutional Review Board of Juntendo University Faculty of Medicine, Tokyo, Japan (receipt number 813; approval letter number 2012057; May 21, 2012).

### Data collection

#### Questionnaire for exposure and covariate assessment

Participants completed a self-administered questionnaire collecting information on socio-demographic and anthropometric characteristics, lifestyle including exercise habits, and medical history including hypertension, type 2 diabetes, and cardiovascular disease. BMI was calculated as weight in kilograms divided by the square of height in meters. Participants were also asked to recall their body weight at approximately 20 years of age. Adult weight gain was then estimated by deducting this recalled weight from their current body weight. The revised version of the International Physical Activity Questionnaire (IPAQ)–Short Form Japanese edition was used to evaluate individual levels of physical activity in terms of intensity and frequency in a usual week (MET-h/week) [9, 10]. The self-administered IPAQ short version has been validated against accelerometer-measured activity levels among Japanese adult men and women, resulting in correlation coefficients of 0.39 and 0.37 (*P* < 0.001) for the CSA accelerometer and the life coder accelerometer, respectively [9].

#### SDB screening

The number of apnea and hypopnea episodes occurring during one night was measured using a single-channel airflow monitor (SOMNIE; NGK Spark Plug Co. Ltd., Nagoya, Japan) [11, 12] to obtain an individual respiratory disturbance index (RDI). Among healthy-weight people (BMI <25 kg/m^2^), the RDI measured by SOMNIE was highly correlated with the apnea–hypopnea index as assessed by polysomnography (*r* = 0.93), and its sensitivity and specificity for predicting an apnea–hypopnea index ≥15 events/h were 0.78 and 0.89, respectively [12].

The recording of airflow during sleep was carried out using a portable monitoring device in the participants’ homes. Participants were provided with an instruction leaflet for the portable monitor and a sleep log. The leaflet instructions read as follows: “The recording should be carried out for more than 4 h. Please engage in your normal life at home, including diet, drinking, smoking, and taking medicine.” Among night shift workers, 17 participants conducted the recording during sleep after a day shift, 6 after a night shift, and 29 during a day off.

Thirteen participants were excluded from the analysis because of imprecise (n = 9) or missing RDI values (n = 4) caused by poor measurement conditions. Data from the remaining 180 men with precise RDI values were used for the analysis. An SDB case in the present study was defined as having an RDI of at least 15 apneas/hypopneas/h, as in a previous study [5]. Using this criterion, the subjects were divided into two groups: 49 cases and 131 controls.

### Statistical analysis

All statistical analyses were performed using SAS software version 9.2 for Windows (SAS Institute Inc., Cary, NC, USA). There was a BMI limit (<30 kg/m^2^) for inclusion in our analysis, following the World Health Organization’s definition of obesity [13] (three cases and six controls were excluded). Characteristics were compared between cases and controls using the Wilcoxon rank-sum test with normal approximation for continuous variables and the Fisher’s exact probability test for categorical variables. Individual physical activity levels were categorized into tertiles among the control subjects, and corresponding dummy variables were created. Here, tertiles were defined by two cut points that divided the participants into three groups of equal size based on the distribution of physical activity among the controls. Unconditional logistic regression analysis was performed to calculate crude and multivariable-adjusted odds ratios (ORs) and 95% confidence intervals (CIs) for SDB according to tertile of physical activity level using the SAS LOGISTIC procedure. A linear trend was tested in logistic regression models using the median value for each physical activity level tertile. Adjusted models included age as a covariate and then added BMI, education, marital status, history of hypertension, smoking and drinking habits, and night shift work. These variables were selected as potential confounders based on a comparison of characteristics between cases and controls and previous studies [1, 4, 5]. In particular, age and BMI are well-known risk factors for SDB [8]. We did not adjust for adult weight gain, because this may be an intermediate variable. Missing values were managed by complete case analysis, meaning that observations with missing values were not used for the multivariable analyses (listwise deletion). All *P* values and 95% CIs reported were two-sided, and significance was set at *P* < 0.05.

## Results

Table 1 presents the characteristics of case and control subjects. Cases were distinguished by having an RDI of 15 or more episodes/h. The prevalence of SDB was 26.9%. Compared with the control subjects, SDB case subjects were less likely to be leaner or night shift workers, and more likely to be older or cohabiting with their spouses. Most participants were engaged in specialized or technical work, managerial work, or marketing and sales. All participants were regular employees.

**Table 1:** Characteristics of study participants (non-obese men) grouped by respiratory disturbance index (RDI)

	Sleep disordered breathing		*P* ^a^
	Cases	Controls	
	(RDI ≥15)	(RDI <15)	
n	46	125	
RDI [events/h], mean (SD)	24.8 (8.8)	8.4 (3.8)	<0.001
Age [years], mean (SD)	45.4 (7.9)	36.9 (10.0)	<0.001
Body weight [kg], mean (SD)	75.0 (8.1)	67.8 (8.5)	<0.001
Body mass index [kg/m^2^], mean (SD)	25.1 (2.2)	22.9 (2.5)	<0.001
Adult weight gain [kg], mean (SD)	11.4 (5.8)	5.2 (6.6)	<0.001
Physical activity [MET-h/week], median (25%, 75%)	12.6 (6.2, 28.6)	18.1 (8.0, 67.5)	0.095
Overtime hours [h/month], median (25%, 75%)	13.5 (1, 30)	20 (10, 40)	0.19
Sleep duration [h/day], mean (SD)	5.9 (0.9)	6.2 (0.9)	0.055
Pittsburgh Sleep Quality Index (PSQI) score, mean (SD)	5.8 (2.3)	5.3 (2.2)	0.25
Current smoker, n (%)	16 (35)	58 (46)	1.00
Regular drinker, n (%)	37 (80)	83 (67)	0.040
Marital status (cohabiting after marriage), n (%)	30 (65)	60 (48)	0.058
Education (university or higher), n (%)	34 (74)	72 (58)	0.075
Work type, n (%)			0.008
Specialized or technical	5 (11)	28 (23)	
Managerial	20 (43)	24 (20)	
Clerical	0 (0)	5 (4.1)	
Marketing and sales	19 (41)	45 (37)	
Service	1 (2.2)	15 (12)	
Production or labor services	1 (2.2)	2 (1.6)	
Other	0 (0)	4 (3.3)	
Night shift worker, n (%)	6 (13)	43 (35)	0.007
History of hypertension, n (%)	10 (22)	11 (9.2)	0.035
History of type 2 diabetes, n (%)	4 (9.1)	3 (2.6)	0.089
History of cardiovascular disease, n (%)	4 (9.1)	5 (4.2)	0.25

The number of participants varied across variables as a result of missing information

^a^Wilcoxon rank-sum test was used for continuous variables, and Fisher’s exact probability test was used for categorical variables

Table 2 shows the ORs and 95% CIs for SDB by level of physical activity. The unadjusted analysis showed a significant inverse association between physical activity and SDB: crude ORs for the tertiles of physical activity were 1.00 (low), 1.58 (middle), and 0.27 (high) (95% CI 0.08–0.88; *P* for trend = 0.007). However, this association was attenuated after adjusting for age and other covariates: Adjusted ORs were 1.00 (low), 1.65 (middle), and 0.41 (high) (95% CI 0.10–1.61; *P* for trend = 0.11). Similar results were obtained when RDI was dichotomized using 10 rather than 15 as the cutoff point (data not shown). Even when middle and high physical activity groups were combined, no significant association was found (adjusted OR = 1.12; 95% CI 0.45–2.77). In addition, when RDI and physical activity level were included as continuous variables, multiple linear regression analysis did not show any significant association between physical activity and RDI (*P* = 0.69). In this multiple linear regression, the included independent variables were physical activity, age, BMI, smoking, drinking, education, marital status, night shift work, and history of hypertension. This analysis also showed a positive association between BMI and RDI (*P* = 0.015).

**Table 2:** Odds ratios (ORs) and 95% confidence intervals (CIs) of sleep-disordered breathing for three levels of physical activity among non-obese male Japanese workers

	Physical activity tertile (range) [MET-h/week]			
	Low	Middle	High	*P* for trend
	(0–9.65)	(9.9–45.2)	(45.3–376.8)	
Cases (RDI ≥15), n	14	24	4	
Controls (RDI <15), n	36	39	39	
Crude OR (95% CI)	1 (reference)	1.58 (0.71, 3.52)	*0.27 (0.08, 0.88)*	*0.007*
Adjusted OR (95% CI)^a^	1 (reference)	1.65 (0.71, 3.83)	0.41 (0.12, 1.45)	0.075
Adjusted OR (95% CI)^b^	1 (reference)	1.98 (0.82, 4.79)	0.46 (0.13, 1.67)	0.098
Adjusted OR (95% CI)^c^	1 (reference)	1.65 (0.63, 4.31)	0.41 (0.10, 1.61)	0.11

Italics indicates statistically significant values (*P* < 0.05)

Respiratory disturbance index (RDI), dichotomized as ≥15 or <15 episodes/h, was used as a dependent variable

^a^Adjusted for age (continuous)

^b^Adjusted for age (continuous) and body mass index (continuous)

^c^Adjusted for age (continuous), body mass index (continuous), education (junior/technical college or lower, university or higher), marital status (cohabiting after marriage, all other values), history of hypertension (yes, no), current smoker (yes, no), regular drinker (yes, no), and night shift work (yes, no)

## Discussion

This cross-sectional study investigated the association between self-reported physical activity and SDB, adjusting for participants’ characteristics, among non-obese male workers in Japan. The results showed that higher levels of physical activity were not clearly associated with a reduced risk of SDB.

Previous studies have shown inverse associations between physical activity and SDB [1, 4–6]. However, the participants in these studies were predominantly obese or overweight people in the United States. To our knowledge, the present study is the first to investigate the association between physical activity and SDB among non-obese men in an Asian country.

Despite having a small sample and moderate statistical power, our results suggest that physical activity may not mitigate SDB among non-obese male adults in Japan. This non-significant association might be biologically plausible, because, for non-obese people, physical activity may not be expected to further improve or prevent SDB via weight change. Other possible mechanisms, such as increased ventilatory muscle strength and endurance and the redistribution of adipose tissue from the pharyngeal regions to other areas, may not be sufficiently developed by physical activity in the present study. To obtain such effects, a course of aerobic physical training would be needed. For example, a randomized controlled trial showed that 150 min/week of moderate intensity aerobic activity for 12 weeks resulted in a significant reduction of total body fat and apnea–hypopnea index without a significant change in body weight [3]. As a result, for non-obese SDB patients in Japan, treatment with continuous positive airway pressure is a good choice to reduce the risk of complications such as type 2 diabetes, cardiovascular diseases, and possible future traffic accidents despite suboptimal long-term adherence [14]. However, as a matter of course, increasing physical activity is still recommendable because of its preventive effects on many diseases other than SDB [15–21].

Major strengths of the present study were as follows. First, almost all invited subjects participated in the study, reducing the possibility of nonresponse bias. Second, many confounders were measured and statistically controlled through multivariable analysis.

Several potential limitations also warrant mention. First, although the correlations between IPAQ scores and the accelerometer-measured values (*r* = 0.39 and 0.37) were at least as high as those reported in previous studies [9], measurement error in the exposure assessment might have caused some degree of misclassification, leading to a null result. Second, although we adjusted for potential confounders to the extent possible, we could not exclude the possibility of residual confounding by unmeasured confounders. Third, the small sample size used in this study might not have allowed us to detect weak associations, although a significant crude OR was observed in the highest physical activity tertile group. The results presented here should be confirmed through replication by larger studies in the future. Fourth, the generalizability of our findings might be limited, because all participants were men and employed by a single company. However, these participants can be considered typical workers in Japan as far as their physical activity levels and prevalence of SDB are concerned. The prevalence of SDB in the present study (26.9%) was relatively consistent with those reported in surveys of male adults in Japan (22.3%) [22], Australia (24.9%) [23], and Brazil (24.8%) [24], when SDB is defined as apnea–hypopnea index ≥15 events/h. Fifth, although this study investigated the association between physical activity level and SDB among non-obese men, the mechanistic pathway of this association could not be explored because we employed a cross-sectional design and did not measure such mediator variables. In addition, we used a single-channel airflow monitor instead of polysomnography. The use of polysomnography in future studies may reduce the possibility of outcome misclassification.

## Conclusions

In a cross-sectional study of non-obese workers in Japan, we found no significant association between physical activity and SDB. Our results do not suggest that physical activity had a protective effect against the development of SDB among non-obese Japanese men.

## Abbreviations

BMI: body mass index
CI: confidence interval
IPAQ: International Physical Activity Questionnaire
MET: metabolic equivalent
OR: odds ratio
PSQI: Pittsburgh Sleep Quality Index
RDI: respiratory disturbance index
SDB: sleep-disordered breathing
